# 3-Dimensional Modeling Guided Transcatheter Repair of Dehisced Pulmonary Venous Baffle With Gore ASD Device

**DOI:** 10.1016/j.jaccas.2023.101968

**Published:** 2023-08-02

**Authors:** Analise Sulentic, Mudit Gupta, Silvani Amin, Yan Wang, Danish Vaiyani, Patricia Sabin, Sara L. Partington, Matthew J. Gillespie, Matthew A. Jolley

**Affiliations:** a Department of Anesthesiology and Critical Care Medicine, Children’s Hospital of Philadelphia, Philadelphia, Pennsylvania, USA; b Division of Cardiology, Children’s Hospital of Philadelphia, Philadelphia, Pennsylvania, USA; c Department of Medicine, Hospital of the University of Pennsylvania, Philadelphia, Pennsylvania, USA

**Keywords:** 3D echocardiography, 3D modeling, cardiac MR, device occlusion, partial anomalous pulmonary venous return, Warden procedure

## Abstract

A 38-year-old woman with sinus venosus atrial septal defect and partial anomalous return of the right upper pulmonary vein underwent a Warden procedure but experienced a large residual defect after patch dehiscence. Image-derived 3D modeling informed novel device closure with a Gore Cardioform atrial septal occluder. (**Level of Difficulty: Advanced.**)

## Central Illustration

**Figure:**
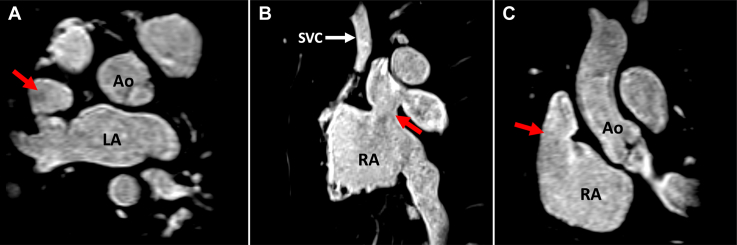

A 38-year-old woman presented with right heart dilation and a residual sinus venosus atrial septal defect (ASD) 1 year after undergoing a Warden procedure for sinus venosus ASD and partial anomalous return of the right upper pulmonary vein (RUPV) to the superior vena cava (SVC). Cardiac magnetic resonance (CMR) demonstrated dehiscence of the patch used to baffle the RUPV flow and a Qp:Qs of 2:1.

**Learning Objectives**

• To better visualize complex defect created by patch dehiscence though multimodality imaging in conjunction with 3D modeling.
• To highlight the novel use of an ASD septal occluder for a dehisced baffle while avoiding surgical repair.

## Medical History

The patient had received a diagnosis of sinus venosus ASD and partial anomalous pulmonary venous return of the RUPV to the SVC at the level of the right pulmonary artery (RPA) during evaluation for palpitations. She underwent a Warden procedure with transection of the SVC above the anomalous RUPV, baffling of the pulmonary venous confluence through the sinus venosus ASD to the left atrium, and reanastomosis of the SVC to the right atrial appendage.

## Differential Diagnosis

The differential diagnosis of right heart dilation includes ASD, anomalous pulmonary venous return, AV valve regurgitation, pulmonary valve regurgitation, pulmonary arterial hypertension, pulmonary embolism, and cardiomyopathy.

## Investigations

**Figure 1:**
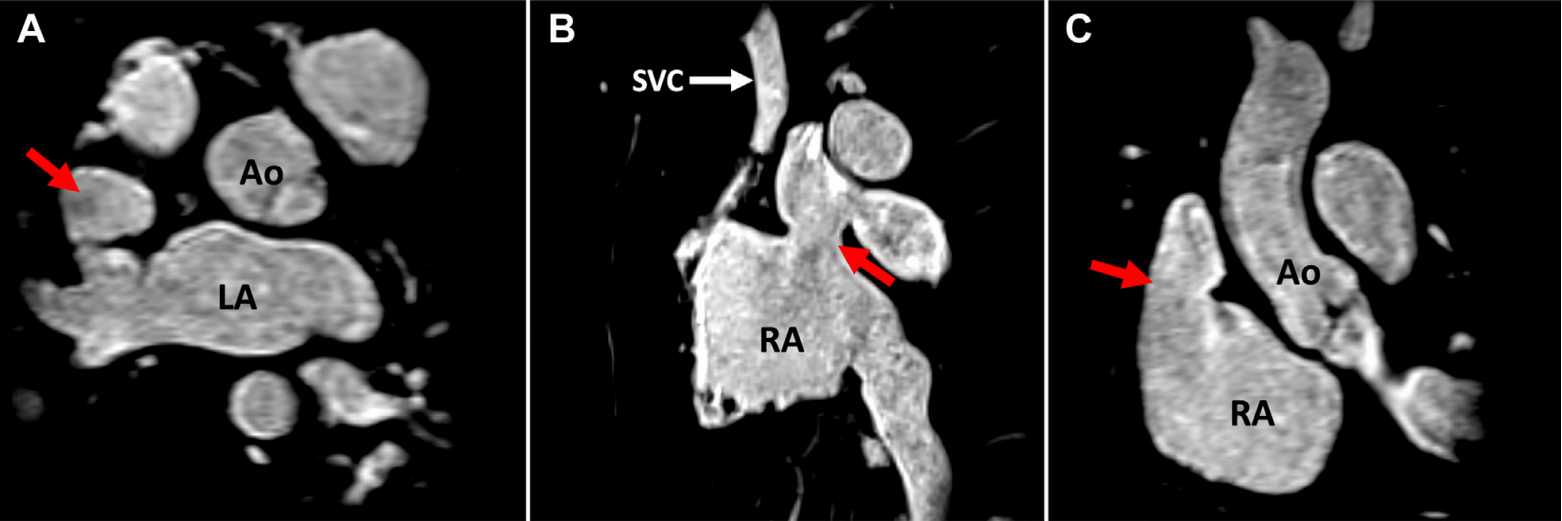
Preprocedural CMR Cardiac magnetic resonance (CMR) demonstrating location of patch dehiscence **(red arrow)** in relation to aorta (Ao) and left atrium (LA), and right atrium (RA) in axial **(A)**, sagittal **(B)**, and coronary **(C)** views.

Approximately 1 year after the Warden procedure, CMR demonstrated that the RUPV and right middle pulmonary vein (RMPV) appropriately returned to the SVC stump, but the autologous pericardial patch used to baffle the pulmonary venous drainage to the left atrium had completely dehisced, with a large residual sinus venous ASD (Figure 1, Video 1). The Qp:Qs by CMR was approximated to be 2:1, with associated moderate dilation of the right ventricle but preserved right ventricle ejection fraction.

**Figure 2:**
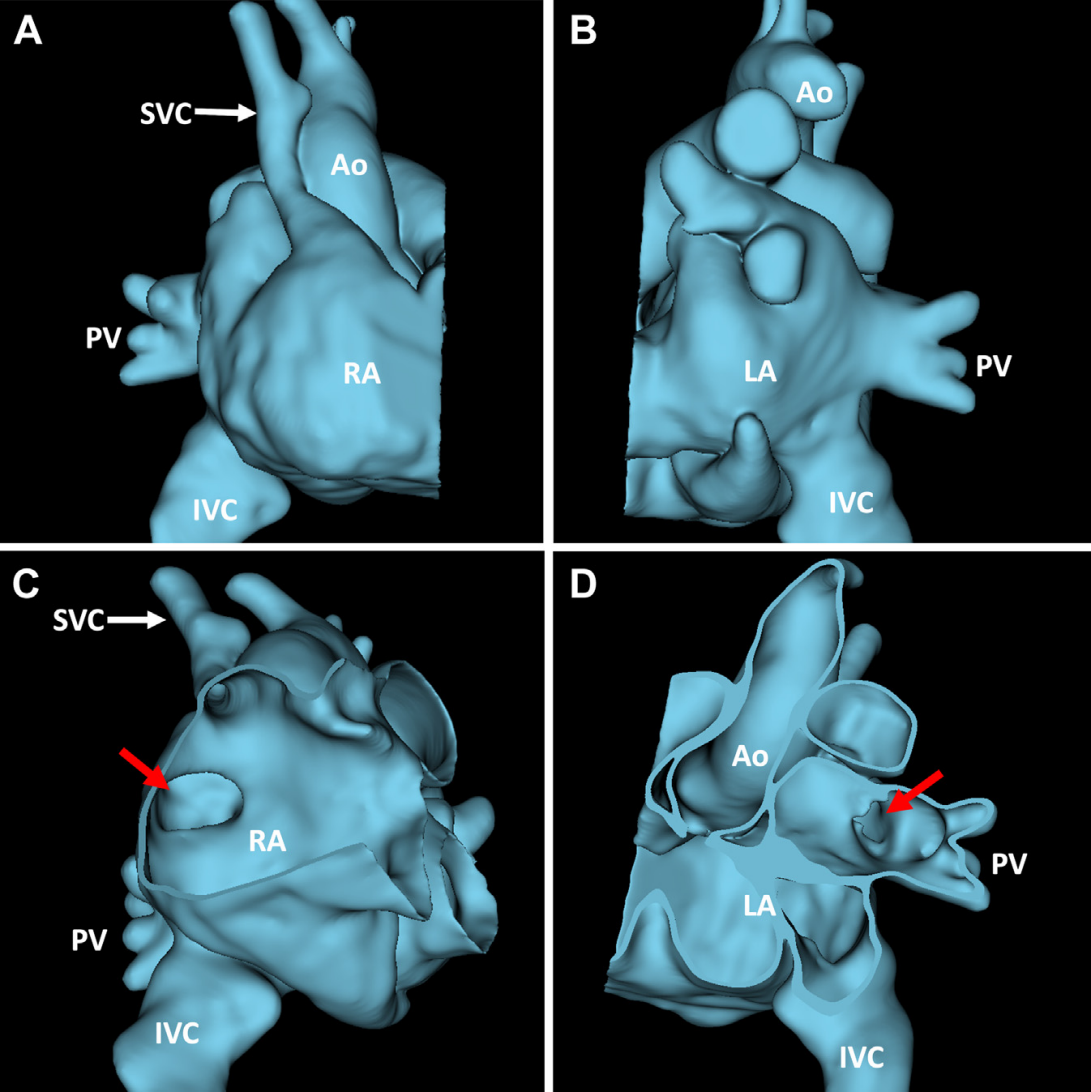
CMR-Based Modeling to Inform 3D Echocardiographic Views **(A)** Right lateral view of heart segmentation. **(B)** Left lateral view of heart segmentation. **(C)** Right lateral view of segmentation displaying site of patch dehiscence **(red arrow)**. **(D)** Left lateral view demonstrating site of patch dehiscence **(red arrow)**. RA = right atrium; LA = left atrium; PV = pulmonary veins; IVC = inferior vena cava Ao = aorta.

**Figure 3:**
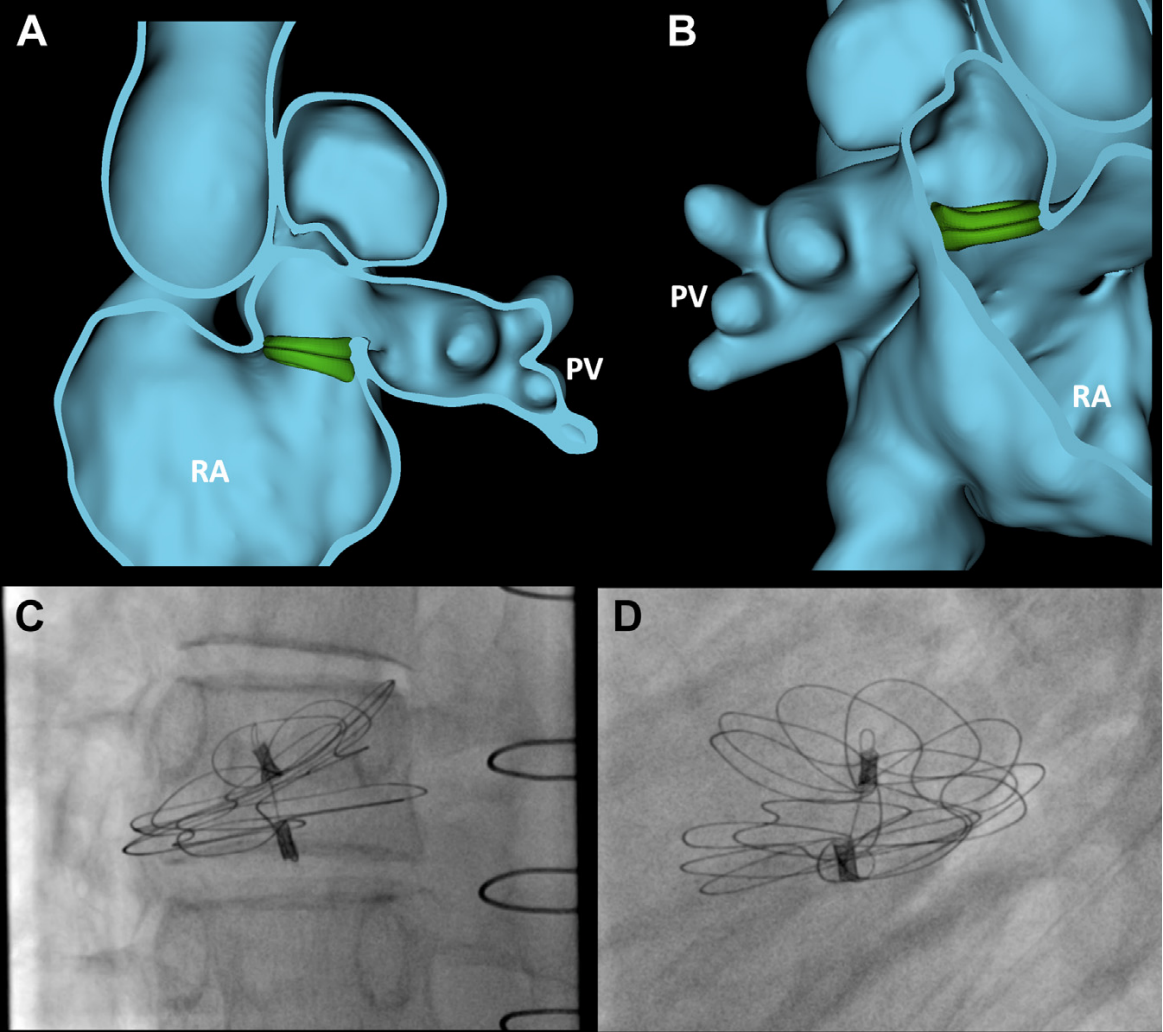
Comparison of Device Simulation With Procedural Fluoroscopy Imaging **(A)** Leftward view of virtual placement of 44-mm device recreates the “floor” of the pulmonary venous baffle. **(B)** Segmentation and device viewed from right lateral perspective. **(C, D)** Analogous angiograms of 44-mm device deployed into defect.

Given the complexity of the structural defect, further preprocedural modeling was performed. The CMR was segmented using the segment editor in the 3D slicer (www.slicer.org) (Figure 2, Video 2).^1^ The segmented model demonstrated a large defect (2.4 × 2.1 cm) in the floor of the SVC stump, allowing RUPV and RMPV flow to drain into the right atrium, as well as rims on the anterior and lateral aspects of the defect. Device closure was conceptualized based on this modeling and then simulated using the cardiac device simulator module in SlicerHeart (Figure 3, Video 3).^1^^,^^2^

## Management

**Figure 4:**
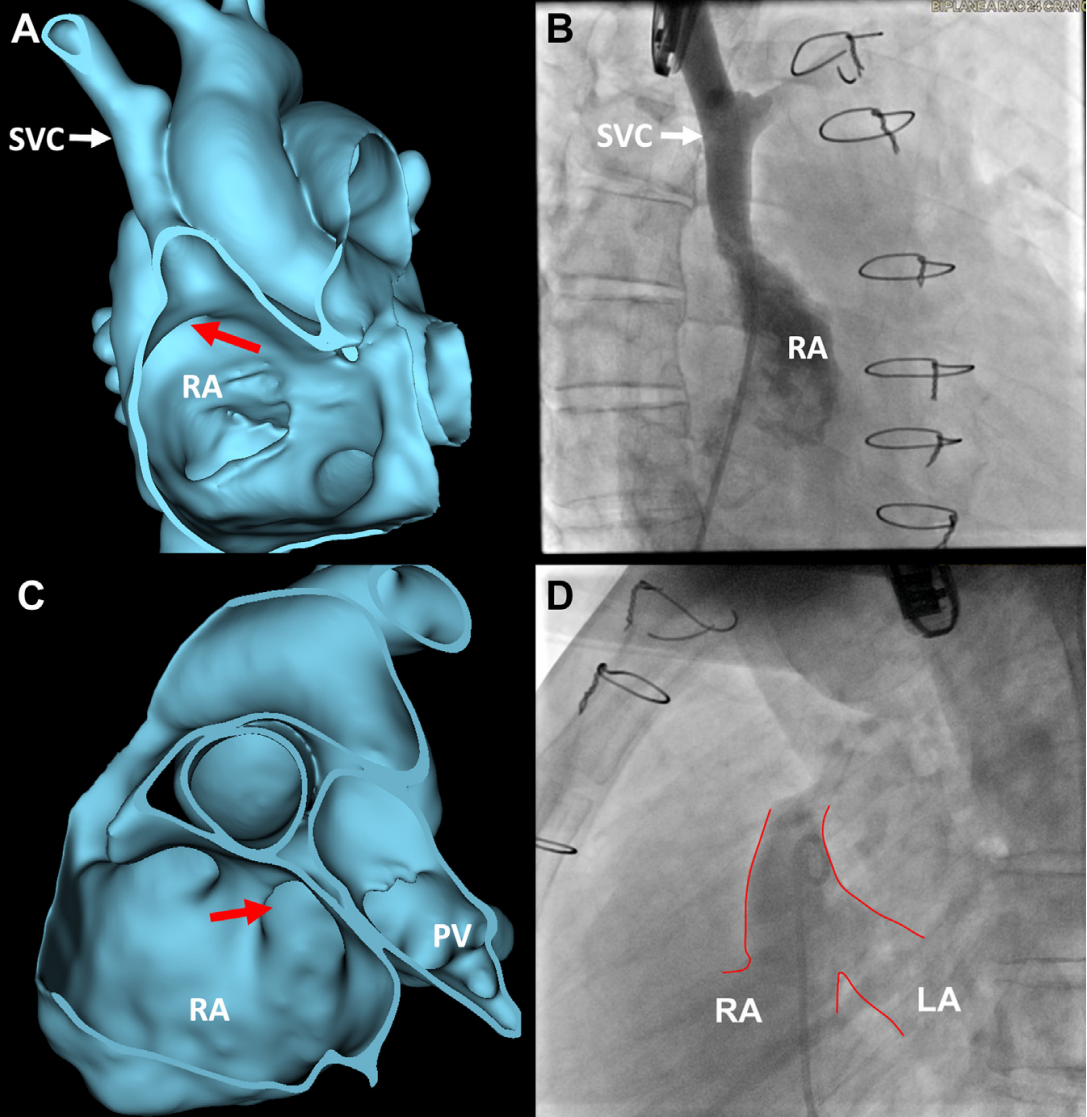
Comparison of Segmentation and Procedural Fluoroscopy Imaging **(A)** Right lateral view of segmentation from right atrium (RA). **Red arrow** highlights defect created by patch dehiscence. **(B)** Angiogram showing right lateral view of contrast material returning to the RA via the SVC status post Warden. **(C)** Segmentation cropped into from an anterior left view with **red arrow** highlighting defect created by patch dehiscence, RA, and pulmonary veins (PV). **(D)** Native SVC stump angiogram displaying pulmonary venous flow draining through Warden baffle into both the left and right atria as a result of patch dehiscence.

**Figure 5:**
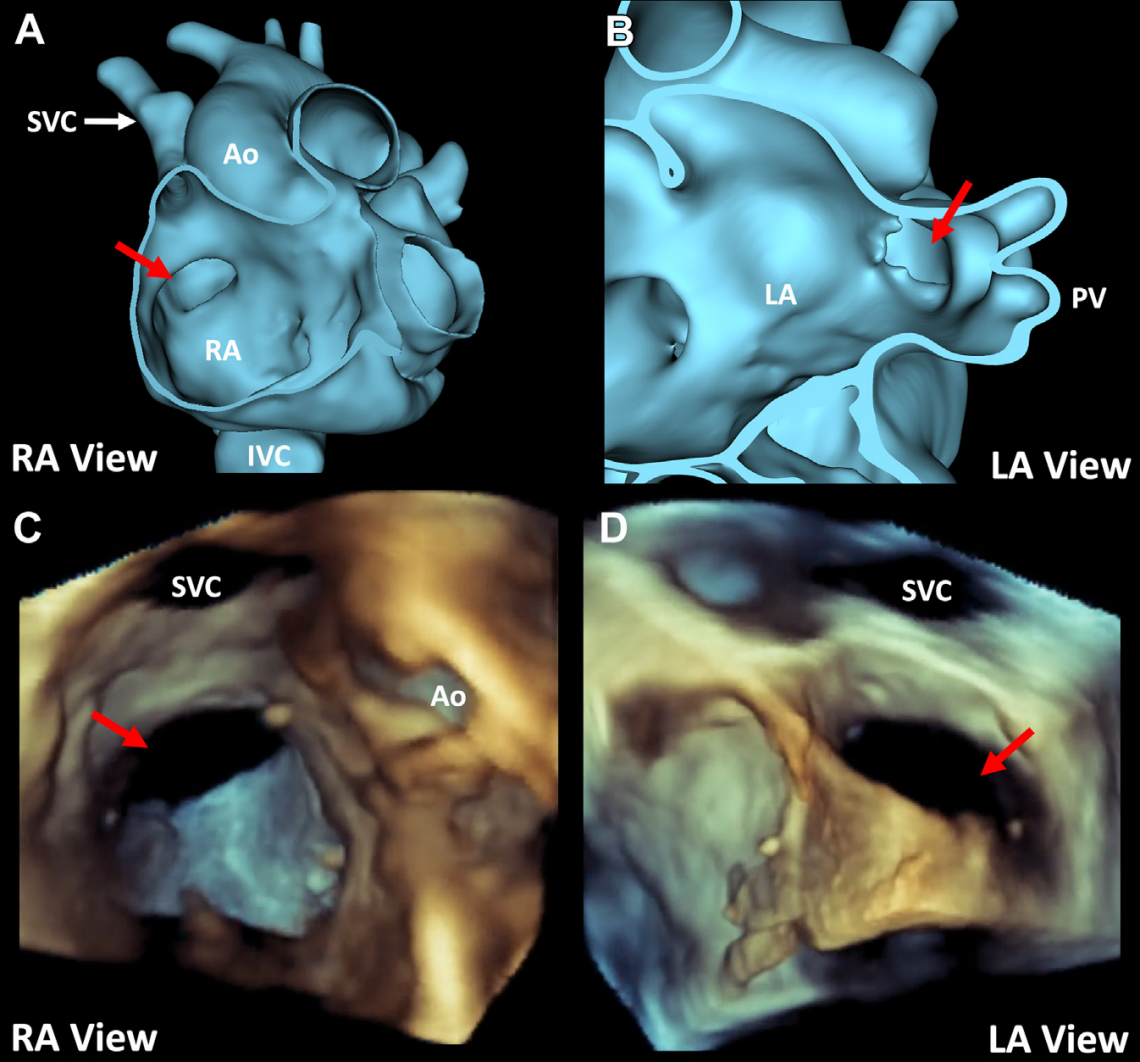
Comparison of Modeling and Procedural 3D TEE **(A)** Segmentation cut into from lateral right perspective. **(B)** Left lateral view of site of patch dehiscence **(red arrow) (C)** 3D transesophageal echocardiogram (TEE) from right atrial (RA) perspective. **(D)** 3D TEE from left atrial view (LA). SVC = superior vena cava; Ao = aorta.

**Figure 6:**
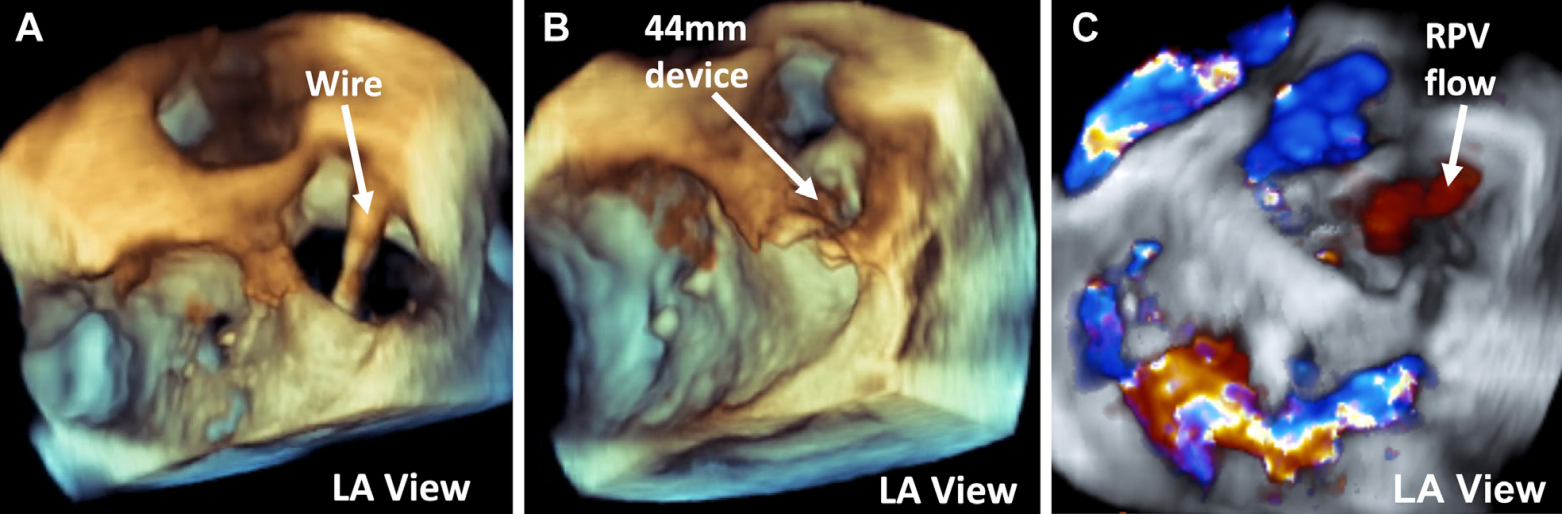
Procedural 3D Transesophageal Echocardiogram **(A)** 3D TEE of wire through defect from left atrial (LA) view. **(B)** 3D TEE with color Doppler of 44-mm device deployed into defect from LA view. **(C)** 3D TEE with color Doppler of 44-mm device deployed into defect without obstruction of the right pulmonary venous (RPV) flow.

After discussion of the risks and benefits of various treatment options, the patient elected to attempt catheter-based intervention as opposed to surgical recreation of the baffle. Procedural transesophageal echocardiography (TEE) and angiography confirmed findings of a large sinus venosus ASD at the floor of the SVC/pulmonary vein stump with left-to-right flow (Figures 4 and 5). Real-time 3D TEE was used almost exclusively for guidance of device placement because of familiarity with the 3D anatomy of the patient. Through a 14-F sheath in the right atrium, a 44-mm Gore Cardioform ASD occluder (Gore Medical) was advanced across the defect and deployed with the “left atrial” disc within the pulmonary venous baffle and the “right atrial” disc within the right atrium across the native inflow of the SVC, thereby re-establishing the floor of the baffle. 3D TEE demonstrated stable device position with minimal residual flow around the device and no obstruction of the right pulmonary vein inflow to the left atrium (Figure 6, Video 4).

## Discussion

The Warden procedure includes surgical repair baffling of the pulmonary venous flow through an atrial level defect into the left atrium in patients with sinus venosus ASD and anomalous right pulmonary vein return. Disruption of the surgical baffle patch results in a large sinus venosus defect and left-to-right shunt at the atrial level. We describe the first known successful transcatheter closure of a residual sinus venosus defect with an ASD occlusion device after Warden patch dehiscence. An understanding of the structure of this unique defect and conceptualization of a transcatheter approach to intervention was informed by patient-specific CMR-derived modeling, visualization, and virtual device placement.^3^, ^4^, ^5^ The familiarity with the 3D anatomy gained from modeling enabled near-exclusive use of 3D (as opposed to 2D) echocardiography to direct device placement, in conjunction with 2D projection angiography, and tactile feedback from the catheter.

Transcatheter closure of sinus venosus defects are particularly challenging, owing to anatomical variability and structural complexity.^6^ Several groups have reported the use of multimodal imaging, including 3D printed models for procedural planning before transcatheter correction of sinus venosus defects with covered stent placement.^5^^,^^7^^,^^8^ Previously described transcatheter closure techniques involve covered stent placement to redirect anomalous pulmonary venous flow to the left atrium while preserving SVC flow to the right atrium.^5^ Postsurgical patch dehiscence adds further complexity because the surgically altered defects can be unpredictable in location and orientation. In this context, creation of a mental model of the structure of the resulting defect can be challenging using traditional 2D reconstructions of 3D images. 3D modeling may provide a valuable tool for visualization of anatomy and conceptualization of access strategy, device selection, and patient candidacy in this situation.^3^ Notably, unlike previously reported methods of redirecting pulmonary venous flow using covered stents, a septal occlusion device requires rims of tissue bordering at least some of the defect to provide a secure anchor for the device retention discs. Circumferential visualization of these rims using CMR-derived modeling was particularly beneficial to the conception and design of this procedure.

## Follow-Up

Transthoracic echocardiography 1 day after the intervention demonstrated no residual leak around the device and normal right ventricular systolic shortening. The patient was discharged 1 day after the procedure and has not reported complications to date.

## Conclusions

In summary, procedure-specific 3D modeling using CMR was successfully used to visualize postsurgical anatomy and to plan a transcatheter closure of a sinus venosus defect using an atrial occlusion device. Patient-specific, image-derived modeling may be informative to the design of unique transcatheter interventions, particularly for atypical and unfamiliar surgically altered anatomy.

## Funding Support and Author Disclosures

This work was supported by NIH 1R01HL153166, the Topolewski Pediatric Valve Center at the Children’s Hospital of Philadelphia (CHOP), and the Topolewski Endowed Chair in Pediatric Cardiology at CHOP. Dr Gillespie is a consultant/proctor for Gore. All other authors have reported that they have no relationships relevant to the contents of this paper to disclose.

## Appendix

Video 1 Standard Procedural Planning Cardiac magnetic resonance (CMR) with red arrow highlighting the defect created by patch dehiscence in axial, sagittal, and coronal views.

Video 2 CMR-Based Interventional Planning The 3D model of the patient’s heart is displayed and rotated with anatomical labels including inferior vena cava (IVC), aorta, superior vena cava (SVC) to right atrial appendage, and pulmonary arteries. The model is cut into from various directions to provide visualization of site of patch dehiscence.

Video 3 3D Measurements and Device Simulation The 3D model shows the defect created from patch dehiscence **(red arrow) f**rom right and left atrial views, and the diameter of the defect is measured. Transcatheter closure of defect is simulated using an ASD occlusion device in 3D Slicer.

Video 4 Intraprocedural TEE Imaging The cardiac magnetic resonance (CMR)—derived 3D model of the patient’s anatomy is shown, and the superior vena cava (SVC) to right atrial (RA) appendage and site of patch dehiscence (red arrow) is seen from right and left atrial views. Procedural 3D TEE from both the right and left atrial views indicates no residual leak and no obstruction of pulmonary veins after deployment of 44-mm device.
